# Effect of traditional Chinese exercise combined with massage on pain and disability in patients with lumbar disc herniation: A multi-center, randomized, controlled, assessor-blinded clinical trial

**DOI:** 10.3389/fneur.2022.952346

**Published:** 2022-09-06

**Authors:** Xin Zhou, Lingjun Kong, Jun Ren, Pengfei Song, Zhiwei Wu, Tianxiang He, Zhizhen Lv, Shuaipan Zhang, Wuquan Sun, Jiafu Zhang, Junhao Cai, Qingguang Zhu, Min Fang

**Affiliations:** ^1^Yueyang Hospital of Integrated Traditional Chinese and Western Medicine, Shanghai University of Traditional Chinese Medicine, Shanghai, China; ^2^Institute of Tuina, Shanghai Institute of Traditional Chinese Medicine, Shanghai, China; ^3^The Third Clinical School of Medicine, Zhejiang University of Traditional Chinese Medicine, Zhejiang, China; ^4^Shanghai University of Traditional Chinese Medicine, Shanghai, China; ^5^Shanghai First People's Hospital, Shanghai, China; ^6^Shanghai Traditional Chinese Medicine Hospital, Shanghai, China; ^7^Shuguang Hospital, Shanghai University of Traditional Chinese Medicine, Shanghai, China

**Keywords:** lumbar disc herniation, traditional Chinese exercise, traditional Chinese massage, low back pain, disability, lumbar mobility, gait performance

## Abstract

**Background:**

Herniation of the nucleus pulposus caused by disc degeneration and other reasons can cause low back pain and disability. In China, traditional Chinese exercises (TCEs) and traditional Chinese massage (TCM) are widely used to improve symptoms of pain and disability in patients with lumbar disc herniation (LDH). The safety and efficacy of combination therapy have not been studied.

**Objectives:**

To assess the effect of traditional Chinese exercise combined with massage vs. traditional Chinese massage alone on pain, disability, lumbar mobility and gait performance in patients with LDH.

**Methods:**

Multi-center, randomized clinical trial conducted at 4 hospitals in China and enrolling 272 patients with LDH. Participants were randomly assigned to TCEs plus TCM group or TCM alone group. The combined therapy group received 18 Tai Chi training sessions (30-min sessions 3 times a week) and regular TCM treatments over 6 weeks. The control group received TCM therapy alone and was instructed to maintain their usual daily physical activity. Outcome variables measured included Visual Analog Scale (VAS), Short Form of McGill Pain Questionnaire (SF-MPQ), Oswestry Disability Index (ODI), lumbar spine range of motion (ROM) and gait performance.

**Results:**

Among the 272 randomized participants, 259 completed the study. The mean VAS score was 51.77 mm at baseline in the TCEs plus TCM group, and 50.93 mm for the TCM alone group. The reduction in the VAS score at week 6 was greater in the TC group than in the TCM group with a mean difference of 4.05 (95% CI, 2.15–5.95; *P* < 0.001), and the ODI score with between-group differences of 3.57 points (95% CI, 2.84–4.30 points; *P* < 0.001). Similar significantly different results were observed in SF-MPQ, walking speed, cadence, and lumbar ROM. No serious adverse events were reported throughout the study period.

**Conclusion:**

Compared with TCM alone, TCEs combined with TCM treatment performed better in reducing pain and improving disability. The combination therapy could be considered a valuable treatment option for LDH patients, with potential therapeutic utility for middle-aged and elderly patients with LDH.

## Introduction

Lumbar disc herniation (LDH) is caused by partial or complete rupture of the annulus fibrosus due to disc degeneration and other reasons, and the nucleus pulposus protrudes or extrudes from its normal position (1). Typical symptoms of LDH are low back pain (i.e., from annular tears and disc disruption) and leg pain (nerve root irritation or referred pain from degenerative discs) (2). Disc protrusion was found to be the main cause of radicular pain, after lumbar spinal stenosis, spondylolisthesis, and fractures have been excluded (3). In two studies, at least 95% of disc protrusions were at the L4-L5 or L5-S1 level (4, 5). And on imaging, the prevalence of disc protrusion increases with age (from 29% of persons at 20 years of age to 43% of those 80 years of age) (6). The condition of most patients can be improved by conservative therapy, and only a few patients with major neurological deficit or persistent pain after conservative treatment need surgery (3, 5, 7). However, recurrence of pain is common. In a study involving a cohort of patients with sciatica, 25% experienced a recurrence of symptoms within 1 year (8).

Poor quality of life and high disability predict high social costs in patients with chronic low back pain (eg, health care or diminished productivity) (9), especially for elderly group, and this has become an important public health problem worldwide (10). In China, traditional Chinese massage (TCM) and traditional Chinese exercises (TCEs) are widely used in the conservative treatment of lumbar disc herniation because of their economical, effective, and easy-to-accept characteristics (11). Among them, TCM is similar to manual therapy, but it mainly presses the surrounding acupoints along the meridians to relax the spasm muscles. While TCEs include Tai Chi (TC), Yi Jinjing, Baduan Jin, etc. They are moderate-intensity exercises that takes into account the body and mind, emphasizing the coordination and unity of breathing and body movements under the guidance of consciousness (12). Research evidence demonstrated that practicing TC on a regular program can improve the pain and disability (13, 14). In particular, TC has a superior effect in pain and symptomatic management (15). However, analysis of gait patterns in subjects affected by LDH has received little attention in the literature. Few studies have used different techniques (clinical assessment, 3D motion analysis) and experimental conditions (activities of daily living and ground walking) (16–19). In one study, Veronica et al. (20) observed that low back pain patients had lower activity levels with shorter gait speed and time spent in standing position, and longer rest periods compared to healthy subjects.

Therefore, we screened out four typical and easy-to-practice TC movements from common TCEs through the expert Delphi questionnaire (21), and studied the biomechanical characteristics of the lower extremities of these four selected TC movements, and found that the four TC movements have high joint range of motion, slow and strong muscle activity indicated by joint moments which suggest that they are suitable for elderly patients to improve their muscle strength and functional ability (22). While this study hopes to combine TCEs and TCM to summarize superior treatments, and wondered whether this combination therapy may also have a favorable effect on pain and disability in patients with LDH. A more extensive confirmatory study was conducted through clinical assessment and three-dimensional (3D) motion analysis to confirm the efficacy and safety of TCEs combined with TCM in the treatment of LDH. We conducted a multicenter, randomized, controlled, evaluation-blind clinical trial to evaluate the efficacy and safety of combination therapy vs. TCM alone.

## Materials and methods

### Participants

A total of 270 patients diagnosed with LDH were recruited for this study. These included: 90 patients visited Yueyang Hospital of Integrated Traditional Chinese and Western Medicine affiliated to Shanghai University of Traditional Chinese Medicine (YHITCWMSUTCM), and 60 patients each who visited Shanghai First People's Hospital (SFPH), Shuguang Hospital affiliated to Shanghai University of Traditional Chinese Medicine (SHSUTCM) and Shanghai Traditional Chinese Medicine Hospital (STCMH). Socio-demographic variables such as age, gender, weight, height, etc. were obtained from detailed clinical interviews and medical records. The inclusion criteria are as follows: (1) between the ages of 20 and 60, (2) a diagnosis of LDH and present with symptoms such as low back pain, radiating pain, paresthesia or weakness in the lower extremities, (3) history of recurrent low back and leg pain for more than 3 months, (4) score over 30 mm on Visual Analog Scale (VAS) at screening. The diagnosis of LDH is based on both physical examination and the findings of computed tomography (CT) or magnetic resonance imaging (MRI). The exclusion criteria included: (1) history of severe spinal trauma pathology such as spinal bone tumor, tuberculosis, osteoporosis, spondylolisthesis and compression fracture, (2) had previous spinal surgery due to neurological deficits or cauda equine syndrome, (3) patients with serious diseases or mental illness such as cardiovascular, cerebrovascular, hematopoietic and digestive system, (4) had other autoimmune diseases, allergic diseases and acute and chronic infections, (5) pregnant women, (6) MRI examinations should not be performed, such as those with pacemakers, neurostimulators, metal foreign bodies and insulin pumps, (7) participate in other clinical trials within 3 months.

The included disc herniation had to be restricted to the two levels of protruded or extruded, types of bulging disc, sequestration of the herniated disc, and intravertebral herniation (Schmorl node) were excluded. We evaluated the following disc changes: displaced disc material extending beyond <25% of the disc space, or when no continuity exists between the disc material beyond the disc space and that within the disc space (1). The discs were classified independently by two observers (treating physician/radiologist). When there was disagreement, a third observer classified the images and the outcome was decided by a simple majority.

### Study design

This multi-center, randomized, controlled, assessor-blinded clinical study was conducted in 4 hospitals in China. This study protocol was approved by the Institutional review boards of YHITCWMSUTCM (2016-066), SFPH (2016KY153), SHSUTCM (2016-kykt-22) and STCMH (2016SHL-KYYS-19), and was registered at the Chinese Clinical Trial Registry (ChiCTR-INR-16009455). All participants received a full explanation of this study's protocol and provided written informed consent before study enrollment.

### Randomization and masking

Eligible participants were randomly assigned to receive either traditional Chinese exercises combined with massage (*n* = 135, 45 in YHITCWMSUTCM and 30 in three other hospitals) or TCM alone (*n* = 135, 45 in YHITCWMSUTCM and 30 in three other hospitals) through the central randomization system in a 1:1 ratio. Randomization was stratified by enrollment site in a block size of 4. The random number table was generated by a statistician independent from this study. Participants, outcome assessors, and statisticians were blinded to treatment assignment.

Additionally, all investigators were divided into groups and placed in charge of the intervention procedures, outcome assessment, data management, and statistical analysis. The trial manager was responsible for arranging remote randomization and informing the participants and therapists of intervention allocation. Due to the different intervention procedures in the two groups, participants and intervention practitioners could not be blinded. However, outcome assessors and data curators were blinded to the assignment status of all participants. Each intervention physician performed only one of the two interventions and was not involved in outcome measures.

### Interventions

Both the experimental group and the control group were required to perform TCM therapy three times a week for 6 weeks. Participants in the TCEs plus TCM group were taught 4 simplified and typical TC movements for 6 weeks by experienced certified TC instructors.

### TCEs combined with TCM group

**Figure 1:**
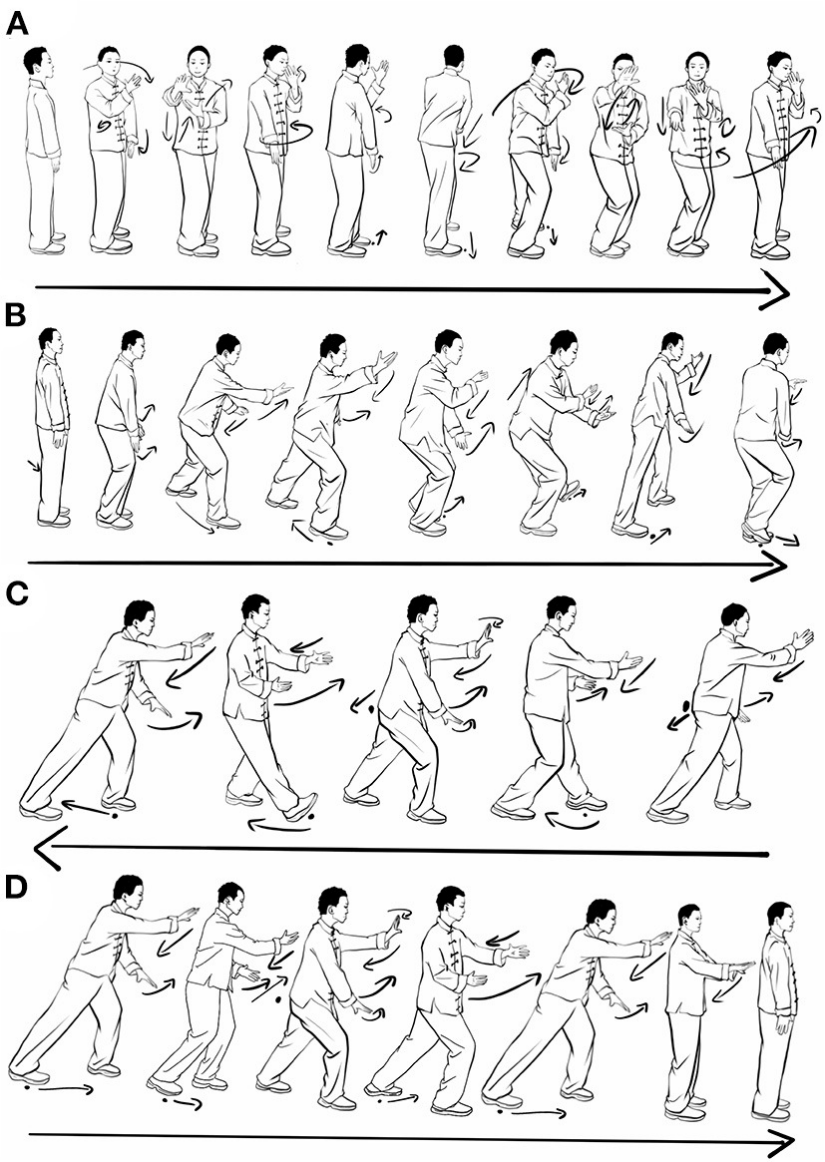
Pictorial representation of 4 TC movements. **(A)** Wave hand in the cloud, **(B)** Leaning fly side, **(C)** Repulse monkey, **(D)** Brush knee twist step.

Participants in the experimental group received 4 typical TC movements exercise training which are included in each TC style (eg, Chen, Yang, Sun, Wu). The 4 TC movements have been simplified by experienced masters, which are more suitable for the elderly and patients with lumbar disc herniation, and are also easier to learn and remember. The selected TC movements are (1) wave hand in the cloud (WHIC), (2) leaning fly side (LFS), (3) repulse monkey (RM), (4) brush knee twist step (BKTS) (Figure 1). Figure 1A shows the WHIC movement which mainly moves in the lateral directions, the movement involves synchronized waving of hands and lateral stepping. Figure 1B illustrates the movement of LFS, which moves mainly in the diagonal direction. Figure 1C indicates the movement of RM, which includes backward stepping while pushing the same palm forward. Figure 1D is the BKTS movement that moving forward, both BKTS and RM were performed inline but in opposite directions.

Before directing the intervention, a treatment leader trained the therapists at each center until they were deemed competent, and then provided regular individual supervision to the participants. Each participant received guidance intervention three times a week, each lasting 30 min. The first session occurred within 3 business days of randomization and was delivered face to face, *via* Wechat, or by telephone. Up to 3 further appointments of 20 min each were offered over 6 weeks, and participants could choose to receive the guidance either by Wechat or telephone. In addition, participants were given a self-exercise booklet and video CD, which detailed the main points of each movement, the duration of physical activity, and the intensity. The guidance intervention lasted 6 weeks with 18 sessions, and during each session, the mentors also answered questions and concerns raised by participants about each exercise. Furthermore, the therapist asks about progress, with an emphasis on advancing to the next step. They recognize trainee achievements and provide feedback on trainee efforts to improve trainee motivation and self-efficacy. Participants were asked to practice for at least 20 min per day on days without training sessions and to keep a personal log of daily practice. Importantly, if participants found that symptoms increased after a gradual change in their activity levels, they were advised to keep their activity levels at the same level for more than a week until symptoms resolved before considering increasing their activity levels again.

**Figure 2:**
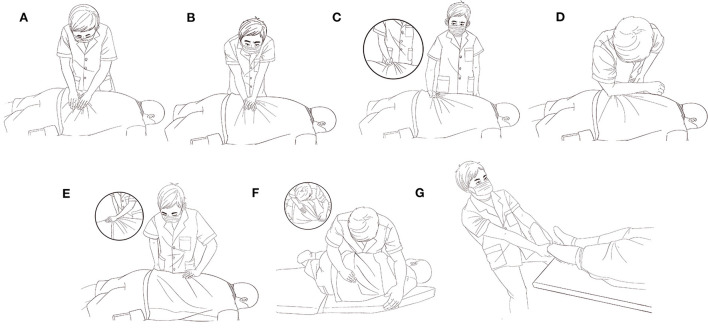
Pictorial representation of TCM intervention. **(A)** Fingers pressing, **(B)** kneading, **(C)** Rolling, **(D)** Elbow pressing, **(E)** Pushing, **(F)** Spinal manipulation, **(G)** Stretching.

Meanwhile, participants received 20 min of TCM therapy three times a week. During the treatment, the subject is required to lie prone, and the therapist first uses the pressing (Figure 2A), kneading (Figure 2B), and rolling (Figure 2C) techniques to relax the erector spinae, multifidus, gluteus medius, gluteus maximus, hamstrings, and gastrocnemius muscles along the bladder meridian on both sides of the spine for about 12 min. Then use fingers or the tip of the elbow to press the bilateral acupoints (BL23, BL24, BL25, BL39, BL54, BL57, GB29, GB30), as well as pain points and spasms muscle for about 5 min (Figure 2D). Finally, pushing along the waist to the lower extremity to relax the muscles (Figure 2E), and performing spinal manipulation to adjust the joints, the practitioner usually entail a rotational thrust to the lumbar vertebral column whilst distraction is applied along its length (Figure 2F), and then stretches the lower limbs for a total of 3 min (Figure 2G).

### Control group

Participants received only the TCM intervention and were asked to maintain their usual daily physical activity, but the group was not assigned any exercises or specific behavior management training.

The number of contacts with the physical therapist during the trial was recorded, as well as the pattern and duration of each contact. The therapists were responsible for following the participants during the trial. Participants were assessed at baseline and 6 weeks after randomization, with trial managers conducting baseline assessments face-to-face in the clinic or by telephone. All measurements were self-assessed by participants.

### Outcome measures

Outcome assessments included questionnaires administered directly at baseline (before randomization) and after treatment (week 6). Participants were not blinded to treatment, which precluded blinding of assessments of self-reported outcomes.

### Primary outcome measure

The primary outcomes were participants' VAS score and Oswestry Disability Index (ODI) at baseline and follow-up after the 6-week treatment. VAS was assessed on a 100 mm horizontal line and subjective pain perception was quantified on a scale from 0 to 100, with 0 representing no pain and 100 being the worst pain imaginable. The ODI needs participants to assess how their leg and back pain affected nine activities: personal care, weightlifting, walking, sitting, standing, sleep, employment/housework, travel, and social life. The answer to the first question is according to the necessity of painkiller intake. Each answer is rated from 0 to 5. Based on the total score (ranging from 0 to 50), disability in to evaluate disability for LDH as minimal, moderate, severe, crippling back pain, or disability that makes the patient bed-bound (23). ODI score ≤ 22 can be used as a criterion for successful treatment of patients with lumbar spine disease (24).

### Secondary outcome measure

The secondary outcome measured for all of the participants was the Short Form of McGill Pain Questionnaire (SF-MPQ). Participants recruited at the main center were also assessed by 3D motion capture for gait performance and lumbar flexion angle.

### SF-MPQ

The SF-MPQ consists of Pain Rating Index (PRI), Current Pain Intensity (PPI), and VAS. For PRI, subjects were asked to describe the sensory and emotional qualities they experienced. Descriptors are rated on an intensity scale of 0 = none, 1 = mild, 2 = moderate, or 3 = severe. PRI is the sum of the intensity values of descriptors that characterize pain. The PPI is graded on six scales from 0 (no pain) to 5 (extremely painful).

### Gait performances and lumbar spine activity

3D kinematics data were sampled using 15 VICON Vero cameras (Oxford Metrics, Oxford, UK) at 100 Hz and two force plates recorded at 1,000 Hz (BMS400600, AMTI, OH, USA). The force plates were embedded in the middle of the ground of a 6-m-long walkway.

The participant's anthropometric data were measured before recording the movements. Thirty-nine reflective markers attached to skin or clothing with double-sided tape to indicate anatomical landmarks of the body by the Plug-in-Gait marker set (Oxford Metrics), modified from the Helen Hayes marker set (25). The movements were performed while the participants wore their preferred shoes. Before data collection, participants had time to familiarize themselves with the experimental environment. Record at least five trials of both maximum flexion of the lumbar spine and walking at the participant's preferred comfortable speed. Participants were instructed to touch the force plate with only one foot at a time while walking.

Adverse events were based on vital signs, physical examination, and other test results assessed at each visit. Causal relationships between adverse events and the intervention were assessed, as well as the severity of intervention-related adverse events, such as pain, bleeding, hematoma, or bruising. All outcome values were recorded on a case report form (CRF), designed by the Institute of TCM, Shanghai Academy of Traditional Chinese Medicine, and only accessible to the blinded researchers.

### Data processing and analysis

The 3D joint angles of the lumbar spine and gait parameters were obtained by using VICON Nexus (Oxford Metrics) and the Plug-in-Gait model (Oxford Metrics). A successful gait cycle was defined as walking from foot strike to foot strike on the same foot. Heel strike and toe off were defined by software readouts as the foot contacts and leaves the force plates and by visual inspection of the virtual heel and toe markers locations. Lumbar flexion and extension were defined based on the vertical axis displacement of the C7 marker (26). The range of motion in flexion and extension was the angle between the sagittal thorax axis and the sagittal pelvis axis around the fixed transverse axis of the pelvis. A positive (flexion) angle value corresponds to the situation in which the thorax is tilted forward.

### Statistical analysis

Based on our previous work in LDH, with anticipated ρ2 = 0.90 (13.2% more explanation for clinical benefit than the TCM alone treatment effect correlation model), G*Power (v 3.1.9.2) estimated a minimum sample size of 121 (power of 0.80 and 0.05 error probability). With the expected dropout rate of 10% (e.g., voluntary patient dropout, motion artifacts, etc.), a sample size of 135 participants per group was required.

All statistical analyses were performed using SPSS 21.0 software (SPSS Inc., Chicago, IL, United States) by a statistician who was blinded to the participant's allocation. Descriptive statistics were generated for all variables, and variable distributions are expressed as the means ± standard deviations or as numbers. The chi-squared or Fisher's exact test for categorical variables was used to examine the potential differences in the baseline demographics (sex ratio) and medical history variables between the two groups. For other continuous variables (age, weight, height, and observation indicators), comparisons between treatment groups were assessed using the independent samples *t*-test or Wilcoxon rank-sum test as appropriate and using the Q-Q plot and Shapiro-Wilk test to verify the normality of the data. Statistical significance was set at *p* < 0.05.

## Results

### Clinic assessment

**Figure 3:**
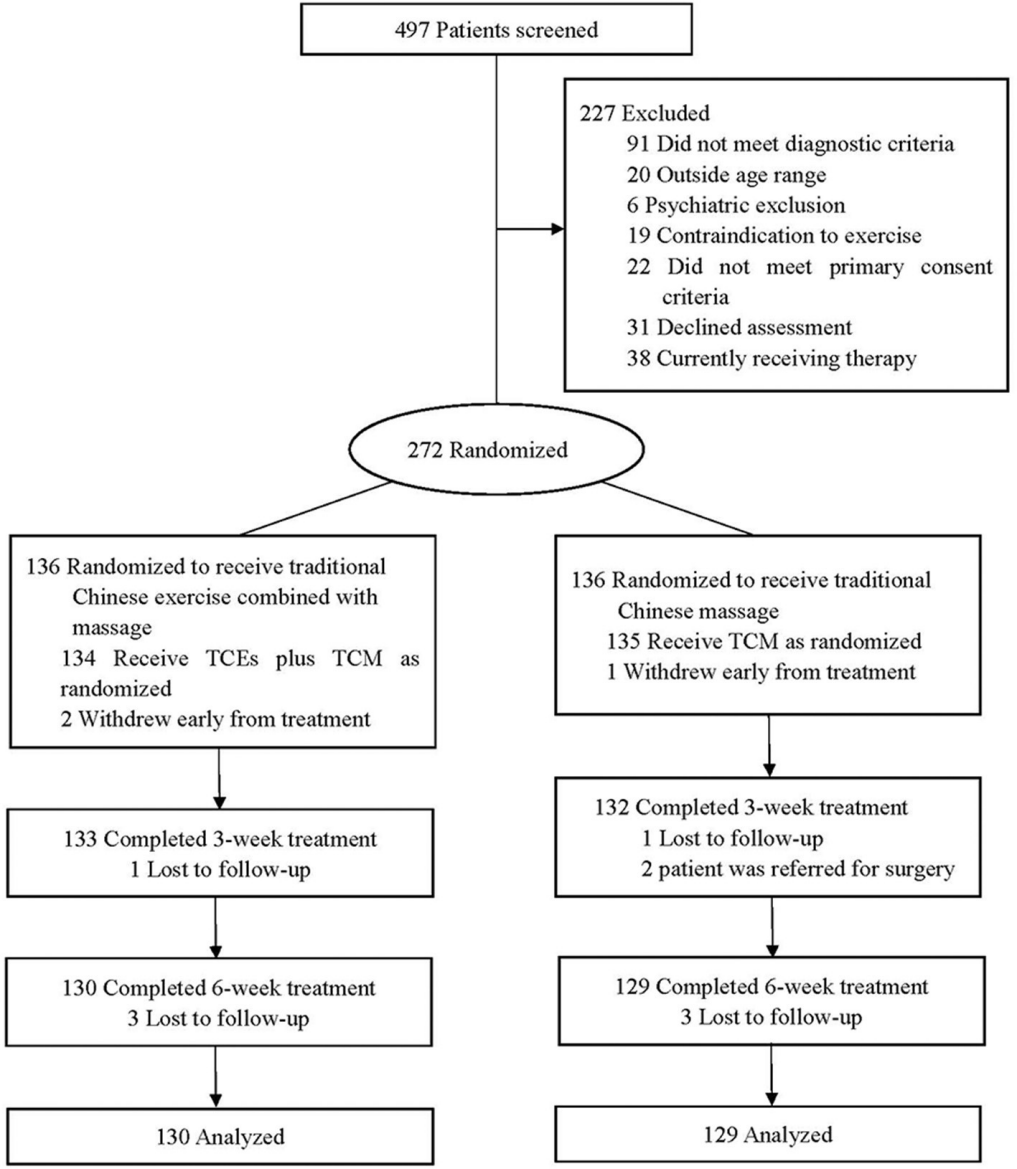
Flow chart of the study design.

Between 5 January 2018, and 30 April 2019, we screened 497 participants for eligibility, of whom 272 were randomly assigned to receive either TCEs plus TCM (*n* = 136) or TCM alone (*n* = 136). Among the randomized participants, 259 (95.2%) completed the study (Figure 3). No serious adverse events were reported throughout the study period. Baseline demographic characteristics and outcome measures at the pre-intervention were similar between the two groups (Table 1). Most participants in the TC group attended more than 9 guidance sessions with the therapist, and most of the participants were satisfied with the TC (Table 2).

**Table 1:** Baseline demographic characteristics of participants (*N* = 259).

	**TCM+TC group (*n* = 130)**	**TCM group (*n* = 129)**	***P Value***
Age (years)	44.36 (10.44)	51.77 (10.32)	0.791
Gender (female/male)	66/64	71/58	0.491
Height (cm)	169.31 (7.96)	168.60 (7.95)	0.475
Weight (kg)	69.66 (11.86)	68.63 (13.46)	0.175
Body mass index (BMI, kg/m^2^)	24.17 (2.89)	24.06 (3.53)	0.230
**Number (%) of previous episodes of low back pain:** ^a^			
None	9 (6.9)	12 (9.3)	0.782
1–5	58 (44.6)	56 (43.4)	
>5	63 (48.5)	61 (47.3)	
Presence of leg pain (%)	98 (75.4)	92 (71.3)	0.459
**Duration (%) of symptoms**			
3–6 months	16 (12.3)	21 (16.3)	0.564
6–12 months	33 (25.4)	35 (27.1)	
>12 months	81 (62.3)	73 (56.6)	
**Level (%) of the herniation**			
L4/L5	24 (18.5)	32 (24.8)	0.457
L5/S1	60 (46.1)	56 (43.4)	
L4/L5 and L5/S1	46 (35.4)	41 (31.8)	
**Drugs for low back pain in past 4 weeks**			
Yes	77 (59.2)	68 (52.7)	0.291
No	53 (40.8)	61 (47.3)	
VAS	51.77 (10.32)	50.93 (10.78)	0.523
ODI	27.44 (6.33)	27.09 (6.20)	0.658

Data are expressed as mean (standard deviation) unless otherwise indicated.

a Period of low back pain separated by at least 3 months of being pain free.

**Table 2:** Mode and attendance at TC sessions and satisfaction with TC (*N* = 130).

	**TCM+TC group**
**First guidance session**	
Face to face	108 (83.1)
Wechat or telephone	22 (16.9)
**Number of guidance sessions attended**	
1–4	9 (6.9)
5–8	14 (10.8)
9–12	22 (16.9)
>12	85 (65.4)
**Satisfaction with TC (*****n*** **=** **118)**^a^	
Moderately or very satisfied	107 (90.7)
Minimally satisfied or dissatisfied	10 (8.5)
Moderately or very dissatisfied	1 (0.8)

Data are number (%).

a The remaining 12 patients did not answer the question.

For the primary outcome, the mean (SD) VAS score was 51.77 (10.32) at baseline and 24.65 (11.11) at week 6 in the TCEs plus TCM group, and 50.93 (10.78) at baseline and 27.86 (11.71) at week 6 in the TCM alone group. The reduction in the VAS score at week 6 was greater in the TC group than in the TCM group with a mean difference of 4.05 (95% CI, 2.15-5.95; *P* < 0.001). Similar results were observed in PRI and PPI (Table 3).

**Table 3:** Primary and secondary outcomes.

	**TCM**+**TC group (*****n*** = **130)**			**TCM group (*****n*** = **129)**			**Between-group**	
	**Pre**	**Post**	**Mean difference (95% CI)**	**Pre**	**Post**	**Mean difference (95% CI)**	**Difference (95% CI)**	***P Value***
**Primary outcomes**								
VAS	51.77 (10.32)	24.65 (11.11)	27.12 (24.50, 29.73)^#^	50.93 (10.78)	27.86 (11.71)	23.06 (20.30, 25.82)^#^	4.05 (2.15, 5.95)	<0.001^*^
ODI	27.44	14.93	12.51	27.09	18.15	8.94	3.57	<0.001^*^
	(6.33)	(4.57)	(11.16, 13.86)^#^	(6.20)	(5.50)	(7.50, 10.37)^#^	(2.84, 4.30)	
**Secondary outcomes**								
PRI	12.28	6.69	5.59	11.71	7.39	4.31	1.28	<0.001^*^
	(2.68)	(2.20)	(4.99, 6.19)^#^	(3.07)	(2.57)	(3.62, 5.00)^#^	(0.89, 1.67)	
PPI	2.48	1.37	1.12	2.37	1.52	0.85	0.26	0.001^*^
	(0.77)	(0.60)	(0.95, 1.28)^#^	(0.75)	(0.71)	(0.67, 1.03)^#^	(0.11, 0.42)	

Data are expressed as mean (standard deviation).

# Difference within groups in mean change from baseline.

* Significance level (*P* < 0.05).

Compared with the TCM group, the TC group had a greater decrease from baseline in the ODI score with between-group differences of 3.57 points (95% CI, 2.84–4.30 points; *P* < 0.001) at week 6 (Table 3). In this study, rescue medication (non-steroidal anti-inflammatory drugs) was given for unbearably severe back and/or leg pain. However, <3 times pharmacological interventions were allowed during the treatment period, and patients with more than 3 times would be discontinued from the study. A total of 12 people in the TCEs plus TCM group were given drug therapy, while 16 people in the TCM alone group received the drugs, and there was no significant difference between the two groups (Table 4).

**Table 4:** Drugs frequency of rescue medication.

**Drugs frequency**	**TCM+TC group (*n* = 130)**	**TCM group (*n* = 129)**	***P Value***
0	118 (90.77)	113 (87.60)	0.666
1	2 (1.54)	1 (0.77)	
2	7 (5.38)	10 (7.75)	
3	3 (2.31)	5 (3.88)	
>3	0	0	

Data are expressed as number (percentage).

### 3D motion analysis

Eighty-two participants were randomly assigned to receive either TCM combine with TC (*n* = 41) or TCM alone (*n* = 41) and completed 3D motion data collection. Baseline demographic characteristics, gait spatiotemporal, and lumbar spine kinematic parameters at the pre-intervention were similar between the two groups (Table 5).

**Table 5:** Baseline demographic characteristics of participants in biomechanical testing (*N* = 82).

	**TCM+TC group (*n* = 41)**	**TCM group (*n* = 41)**	***P Value***
Age (years)	46.85 (10.59)	46.73 (11.91)	0.961
Gender (female/ male)	22/ 19	20/ 21	0.659
Height (cm)	169.61 (7.81)	170.10 (7.31)	0.771
Weight (kg)	70.41 (11.64)	70.15 (11.99)	0.918
Body mass index (BMI, kg/m^2^)	24.35 (2.98)	24.10 (2.84)	0.597
**Number of previous episodes of low back pain:** ^a^			
None (%)	2 (6.9)	4 (9.3)	0.792
1–5 (%)	20 (44.6)	19 (43.4)	
>5 (%)	19 (48.5)	18 (47.3)	
Presence of leg pain (%)	31 (75.6)	30 (73.2)	0.800
Cadence (steps/min)	107.43 (5.21)	107.50 (7.50)	0.963
Velocity (m/s)	1.14 (0.11)	1.15 (0.12)	0.695

Data are expressed as mean (standard deviation) unless otherwise indicated.

a Period of low back pain separated by at least 3 months of being pain free.

First, the gait spatiotemporal parameters of the left and right limbs of all participants were compared. No significant differences were found between the two limbs, suggesting a symmetrical gait pattern. Subsequently, the data of both sides were pooled.

As for the spatiotemporal parameters, change in the mean cadence and velocity of progression from baseline was significantly greater in the TC group than in the TCM alone group with between-group differences of 2.43 steps/min (95% CI, 0.73–4.27 steps/min; *P* = 0.007) after week 6. The stance phase and stride length were similar in both groups (Table 6).

**Table 6:** Spatio-temporal and kinematic parameters of the study groups.

	**TCM**+**TC group (*****n*** = **130)**			**TCM group (*****n*** = **129)**			**Between-group**	
	**Pre**	**Post**	**Mean difference (95% CI)**	**Pre**	**Post**	**Mean difference (95% CI)**	**Difference (95% CI)**	***P Value***
**Spatio–temporal parameters**														
Stance phase (% gait cycle)	61.16 (2.39)	60.94 (2.29)	0.18	60.75 (2.35)	60.95 (2.10)	−0.28	0.43	0.252
			(−0.90, 1.26)			(−1.24, 0.87)	(−0.31, 1.16)	
Double stance phase (% gait cycle)	23.10 (3.33)	22.36 (3.09)	0.74	22.33	22.20 (2.51)	0.13	0.61	0.269
			(−0.67, 2.15)	(3.05)		(−1.10, 1.36)	(−0.48, 1.69)	
Step length (m)	0.63 (0.06)	0.66	−0.22	0.64 (0.54)	0.65 (0.68)	−0.013	−0.01	0.305
		(0.59)	(−0.48, 0.01)			(−0.39, 0.14)	(−0.03,0.01)	
Cadence (steps/min)	107.43 (5.21)	113.59 (5.89)	−6.16	107.50 (7.50)	111.03 (6.80)	−3.53	−2.43	0.007^*^
			(−8.60, −3.71)^#^			(−6.68, −0.38)^#^	(−4.27, −0.73)	
Velocity (m/s)	1.14 (0.11)	1.24 (0.11)	−0.11	1.15	1.21	−0.06	−0.04	0.023^*^
			(−0.15, −0.06)^#^	(0.12)	(0.12)	(−0.11, −0.01)^#^	(−0.08, −0.01)	
**Spinal joint angle (°)**														
Max Flexion	28.39	52.58	−24.18	29.52	46.00	−16.48	−7.29	0.011^*^
	(13.17)	(13.95)	(−30.14, −18.22)^#^	(11.51)	(9.98)	(−21.22, −11.75)	(−12.87, −1.75)	
Standing phase	−16.23	−15.23	−1.00	−15.36	−15.81	0.45	−1.45	0.331
	(5.04)	(6.17)	(−3.48, 1.48)^#^	(6.13)	(4.77)	(−1.96, 2.87)^#^	(−4.40, 1.50)	
ROM	44.62	67.80	−23.18	44.87	61.80	−16.93	−5.96	0.031^*^
	(12.39)	(13.13)	(−28.79, −17.57)^#^	(9.70)	(10.60)	(−21.40, −12.47)^#^	(−11.20, −0.60)	

Data are expressed as mean (standard deviation).

# Difference within groups in mean change from baseline.

* Significance level (*P* < 0.05).

Lumbar range of motion (ROM) and maximum flexion improved significantly in both groups from baseline to post-treatment. The patients in the experimental group had a mean change in flexion of 24.18 (95% CI, 18.22–30.14; *P* < 0.001) and ROM of 23.18 (95% CI, 17.57–28.79; *P* < 0.001), as measured by a 3D motion capture system. The between-group analysis presented that lumbar ROM 5.96 (95% CI, 0.60–11.20; *P* = 0.031) and maximum flexion 7.29 (95% CI, 1.75–12.87; *P* = 0.011) was improved significantly compared with the TCM alone group after 6 weeks of intervention (Table 6).

## Discussion

The current research study aimed to evaluate the effects of active exercise combined with passive massage therapy in addition to routine physical therapy vs. routine TCM therapy alone on pain, functional disability, range of motion, and gait performance in patients with lumbar radiculopathy. According to between-group analyses, when added to TCM, TC significantly improved pain and disability function compared to TCM alone, and we found that especially in improving disability function, significantly more participants exceeded pre-defined clinically meaningful changes for gait performance, lumbar spine activity, and both after TC plus TCM than after TCM alone.

TC is ancient Chinese art practiced as a graceful series of slow and focused movements accompanied by deep breathing, and systematic reviews suggest that TC as stand-alone or add-on therapy can improve pain and function (27). Regarding the efficacy of TCM, no high-quality systematic review exists. However, a randomized trial of chiropractic manipulation for subacute or chronic “back-related leg pain” showed that manipulation was more effective than home exercise in reducing pain (28). And another randomized controlled trial (RCT) involving patients with acute back pain and sciatica with disc protrusion showed that significantly more patients who received chiropractic manipulation had an absence of pain than those who underwent sham manipulation (27). In terms of safety, lumbar neurological complications, including worsening disc herniation or cauda equina syndrome, had also been rarely reported during treatment (29). Therefore, as manual therapy to reduce muscle spasms and increase joint mobility, massage is most beneficial as a supplement to exercise or education, which is also recommended by the American College of Physicians for non-invasive treatment of radicular or non-radial low back pain.

In clinical practice, we often recommend combining passive massage manipulation with active exercise to treat pain and disability caused by LDH. However, there is still a lack of large-scale RCT research support. Therefore, this study compared the TC plus TCM therapy with the easily accepted TCM alone therapy and quantitatively compared the lumbar ROM and gait patterns in subjects with LDH by biomechanical methods. In this study, it was found that compared with the TCM alone group, the treatment effect of TC plus TCM was significantly better, whether it was reducing the pain score or improving the level of disability, especially in terms of walking speed, cadence, and lumbar ROM. in addition, and the step length and stance duration also showed a certain improvement trend, while the main effect of the TCM alone group was more obvious in reducing pain.

The current findings are consistent with a few previously conducted RCTs showing that TC treatment provides better outcomes than either a waiting list or no TC (14, 30). Moreover, a high-quality trial found that for radicular low back pain, spinal manipulation plus home exercise and advice resulted in greater improvements in leg and back pain at 12 weeks compared to home exercise and advice alone (28). Although the duration of treatment, the nature of the treatment, and the results of the study received by the experimental group and the control group were different from this study. A comparison of these findings with this study suggests a longer duration and different conventional treatments. However, these findings are still comparable to this study, suggesting that the combination of treatments used in this study can achieve the same effect in a shorter period. In addition, the gait performance and lumbar ROM were the additional outcome measures not studied previously.

Compared to pharmacological methods, TC is not costly and may benefit physical function (12), particularly in maintaining the control of balance and reducing the risk of falls in older people (31, 32). Multicomponent TC training can improve the proprioceptive function, muscle strength, and mobility of the trunk and lower limbs (33, 34). In addition, studies have paid attention that people with low back pain have different muscle activation patterns compared with healthy controls (35), and they are more likely to exhibit reduced gluteus medius strength, reduced hip abduction force output (36), and altered in muscle recruitment (35). The instability of the lumbar and pelvic due to the inefficiency of gluteal muscles is also related to the existence of low back pain (37). Therefore, as the age increases, the degeneration of the intervertebral disc and the decline of muscle function in the elderly are both important factors causing low back pain, and TC exercise promotes the coordination of the hip and trunk by strengthening and properly activating the muscles of the lower limbs, and the neuromuscular control would benefit low back pain individuals release pain (38). This may be the reason why the TC plus TCM therapy in this study was safer and more effective than TCM therapy alone.

This study has several advantages over previous studies. It was randomized and few patients crossed over to other treatment regimens. In addition, an independent research assistant collected data, observers of assessments were blinded, interventions were standardized, and funding for the study was publicly available. The choice of MRI criteria for inclusion may also be an advantage. When designing the study, we hoped that patients across centers would be as consistent as possible, and to reduce bias due to different therapists through block randomization.

There are several limitations in the current study. First, due to the limitations of biomechanical equipment, the participants in the three sub-centers could not complete the corresponding collection of lumbar range of motion and gait data, resulting in limited sample size. Second, the 6-week follow-up period after the intervention was insufficient to demonstrate the long-term effects of TCEs combined with TCM. And neither the participant nor the practitioner was blind, which could become a potential bias, such as the expectation of the subject and the reliability of the assigned intervention. However, despite these methodological limitations, this study provides objective and clinically beneficial results for assessing the synergistic effect of combination therapy. Therefore, larger-scale and longer-term follow-up intervention studies are needed in the future to confirm the long-term effects of TCEs plus TCM on pain and disability in patients with LDH. In addition, future interventions are warranted to investigate the effects on long-term quality of life and mood.

## Conclusion

In LDH patients with pain and disability, treatment of subjects with the combination therapy resulted in less pain and increased gait performance and lumbar mobility after 6 weeks compared with TCM treatment alone. TCEs combined with TCM therapy can be considered a valuable treatment option for LDH patients and has potential therapeutic utility in middle-aged and older adults with LDH. Further research is needed to understand the long-term efficacy and mechanism of action of this intervention.

## Data availability statement

The original contributions presented in the study are included in the article/supplementary material, further inquiries can be directed to the corresponding authors.

## Ethics statement

The studies involving human participants were reviewed and approved by Institutional Review Boards of Yueyang Hospital of Integrated Traditional Chinese and Western Medicine Affiliated to Shanghai University of Traditional Chinese Medicine (2016-066), Shanghai First People's Hospital (2016KY153), Shuguang Hospital Affiliated to Shanghai University of Traditional Chinese Medicine (2016-kykt-22), and Shanghai Traditional Chinese Medicine Hospital (2016SHL-KYYS-19). The patients/participants provided their written informed consent to participate in this study.

## Author contributions

XZ, LK, JR, QZ, and MF had full access to the data, were responsible for the study concept and design, and critically revised the manuscript for important intellectual content. ZW, PS, TH, ZL, SZ, WS, JZ, and JC contribute to the acquisition of data which were analyzed and interpreted by XZ, LK, and JR. XZ and LK drafted the manuscript. XZ and JR did the statistical analysis. MF, WS, JZ, and JC provided administrative, technical, and material support. XZ, LK, QZ, and MF supervised the study. MF is the guarantor. All authors read and approved the final manuscript.

## Funding

This work was supported by the project of Shanghai Shenkang Hospital Development Center (No. 16CR1023A), Shanghai Science and Technology Innovation Action Plan (21Y21920301), Shanghai Science and Technology Innovation Action Plan Phospherus Cultivation Project (22YF1449900), and Shanghai Pujiang Program (21PJD071).

## Conflict of interest

The authors declare that the research was conducted in the absence of any commercial or financial relationships that could be construed as a potential conflict of interest.

## Publisher's note

All claims expressed in this article are solely those of the authors and do not necessarily represent those of their affiliated organizations, or those of the publisher, the editors and the reviewers. Any product that may be evaluated in this article, or claim that may be made by its manufacturer, is not guaranteed or endorsed by the publisher.

## References

[1] Fardon DF Williams AL Dohring EJ Murtagh FR Gabriel Rothman SL Sze GK. Lumbar disc nomenclature: Version 2.0: Recommendations of the combined task forces of the North American Spine Society, the American society of spine radiology and the American society of neuroradiology. Spine J. (2014) 14:2525–45. 10.1016/j.spinee.2014.04.022 24768732

[2] Deyo RA Mirza SK. CLINICAL PRACTICE. Herniated lumbar intervertebral disk. N Engl J Med. (2016) 374:1763–72. 10.1056/NEJMcp1512658 27144851

[3] Ropper AH Zafonte RD. Sciatica. N Engl J Med. (2015) 372:1240–8. 10.1056/NEJMra1410151 25806916

[4] Peul WC van Houwelingen HC van den Hout WB Brand R Eekhof JA Tans JT. Surgery vs. prolonged conservative treatment for sciatica. N Engl J Med. (2007) 356:2245–56. 10.1056/NEJMoa064039 17538084

[5] Weinstein JN Tosteson TD Lurie JD Tosteson AN Hanscom B Skinner JS. Surgical vs. nonoperative treatment for lumbar disk herniation: The Spine Patient Outcomes Research Trial (SPORT): a randomized trial. JAMA. (2006) 296:2441–50. 10.1001/jama.296.20.2441 17119140 PMC2553805

[6] Brinjikji W Luetmer PH Comstock B Bresnahan BW Chen LE Deyo RA. Systematic literature review of imaging features of spinal degeneration in asymptomatic populations. Am J Neuroradiol. (2015) 36:811–6. 10.3174/ajnr.A4173 25430861 PMC4464797

[7] Overdevest GM Vleggeert-Lankamp CL Jacobs WC Brand R Koes BW Peul WC. Recovery of motor deficit accompanying sciatica–subgroup analysis of a randomized controlled trial. Spine J. (2014) 14:1817–24. 10.1016/j.spinee.2013.07.456 24200407

[8] Suri P Rainville J Hunter DJ Li L Katz JN. Recurrence of radicular pain or back pain after nonsurgical treatment of symptomatic lumbar disk herniation. Arch Phys Med Rehab. (2012) 93:690–5. 10.1016/j.apmr.2011.11.028 22464091 PMC3866041

[9] Mutubuki EN Luitjens MA Maas ET Huygen F Ostelo R van Tulder MW. Predictive factors of high societal costs among chronic low back pain patients. Euro J Pain. (2020) 24:325–37. 10.1002/ejp.1488 31566839 PMC7003839

[10] Knezevic NN Candido KD Vlaeyen J Van Zundert J Cohen SP. Low back pain. Lancet. (2021) 398:78–92. 10.1016/S0140-6736(21)00733-9 34115979

[11] Tang S Qian X Zhang Y Liu Y. Treating low back pain resulted from lumbar degenerative instability using Chinese Tuina combined with core stability exercises: a randomized controlled trial. Complement Ther Med. (2016) 25:45–50. 10.1016/j.ctim.2016.01.001 27062947

[12] Li JX Hong Y Chan KM. Tai chi: physiological characteristics and beneficial effects on health. Brit J Sport Med. (2001) 35:148–56. 10.1136/bjsm.35.3.148 11375872 PMC1724328

[13] Chou R Deyo R Friedly J Skelly A Hashimoto R Weimer M. Non-pharmacologic therapies for low back pain: a systematic review for an american college of physicians clinical practice guideline. Ann Intern Med. (2017) 166:493–505. 10.7326/M16-2459 28192793

[14] Hall AM Maher CG Lam P Ferreira M Latimer J. Tai chi exercise for treatment of pain and disability in people with persistent low back pain: a randomized controlled trial. Arthrit Care Res. (2011) 63:1576–83. 10.1002/acr.20594 22034119

[15] Zou L Zhang Y Yang L Loprinzi PD Yeung AS Kong J. Are mindful exercises safe and beneficial for treating chronic lower back pain? A systematic review and meta-analysis of randomized controlled trials. J Clin Med. (2019) 8:628–43. 10.3390/jcm8050628 31072005 PMC6571780

[16] Novy DM Simmonds MJ Olson SL Lee CE Jones SC. Physical performance: differences in men and women with and without low back pain. Arch Phys Med Rehabil. (1999) 80:195–208. 10.1016/S0003-9993(99)90121-1 10025497

[17] Lamoth CJC Daffertshofer A Meijer OG Beek PJ. How do persons with chronic low back pain speed up and slow down? Trunk-pelvis coordination and lumbar erector spinae activity during gait. Gait Posture. (2006) 23:230–9. 10.1016/j.gaitpost.2005.02.006 16399520

[18] Lamoth CJ Meijer OG Daffertshofer A Wuisman PI Beek PJ. Effects of chronic low back pain on trunk coordination and back muscle activity during walking: changes in motor control. Eur Spine J. (2006) 15:23–40. 10.1007/s00586-004-0825-y 15864670 PMC3454567

[19] Spenkelink CD Hutten MM Hermens HJ Greitemann BO. Assessment of activities of daily living with an ambulatory monitoring system: a comparative study in patients with chronic low back pain and nonsymptomatic controls. Clin Rehabil. (2002) 16:16–26. 10.1191/0269215502cr463oa 11841065

[20] Cimolin V Vismara L Galli M Zaina F Negrini S Capodaglio P. Effects of obesity and chronic low back pain on gait. J Neuroeng Rehabil. (2011) 8:55. 10.1186/1743-0003-8-55 21943156 PMC3186748

[21] Zhou X Zhu QG Kong LJ Cheng YB Yao F Fang L. Expert investigation and research on the treatment of lumbar disc herniation with spinal manual therapy combined with traditional Chinese exercises. Chin J Rehabil Med. (2021) 36:1001–242. 10.1097/MD.0000000000018781 32000379 PMC7004683

[22] Zhu Q Zhou X Zhang S Fang M Li JX. Joint angles and joint moments of the lower limbs in four typical tai chi movements: consideration for management of knee osteoarthritis. Res Sports Med. (2021) 29:586–92. 10.1080/15438627.2021.1975118 34477036

[23] Mekhail N Levy RM Deer TR Kapural L Li S Amirdelfan K. Long-term safety and efficacy of closed-loop spinal cord stimulation to treat chronic back and leg pain (Evoke): a double-blind, randomized, controlled trial. Lancet Neurol. (2020) 19:123–34. 10.1016/S1474-4422(19)30414-4 31870766

[24] van Hooff ML Mannion AF Staub LP Ostelo RW Fairbank JC. Determination of the oswestry disability index score equivalent to a “satisfactory symptom state” in patients undergoing surgery for degenerative disorders of the lumbar spine-a Spine Tango registry-based study. Spine J. (2016) 16:1221–30. 10.1016/j.spinee.2016.06.010 27343730

[25] Davis R. B. Unpuu S. Tyburski D. Gage J. R. (1991). A gait analysis data collection and reduction technique. Hum Movement Sci. (1991) 10:575–87. 10.1016/0167-9457(91)90046-Z

[26] Enrica P Bull MJA McGregor AH. Spinal segments do not move together predictably during daily activities. Gait Posture. (2018) 1:277–83. 10.1016/j.gaitpost.2018.10.031 30391750 PMC6249993

[27] Santilli V Beghi E Finucci S. Chiropractic manipulation in the treatment of acute back pain and sciatica with disc protrusion: a randomized double-blind clinical trial of active and simulated spinal manipulations. Spine J. (2006) 6:131–7. 10.1016/j.spinee.2005.08.001 16517383

[28] Bronfort G Hondras MA Schulz CA Evans RL Long CR Grimm R. Spinal manipulation and home exercise with advice for subacute and chronic back-related leg pain: a trial with adaptive allocation. Ann Intern Med. (2014) 161:381–91. 10.7326/M14-0006 25222385

[29] Oliphant D. Safety of spinal manipulation in the treatment of lumbar disk herniations: a systematic review and risk assessment. J Manip Physiol Ther. (2004) 27:197–210. 10.1016/j.jmpt.2003.12.023 15129202

[30] Weifen W Muheremu A Chaohui C Wenge L Lei S. Effectiveness of tai chi practice for Non-Specific chronic low back pain on retired athletes: a randomized controlled study. J Musculoskelet Pain. (2013) 21:37–45. 10.3109/10582452.2013.763394

[31] Hong Y Li JX Robinson PD. Balance control, flexibility, and cardiorespiratory fitness among older Tai Chi practitioners. Brit J Sport Med. (2000) 34:29–34. 10.1136/bjsm.34.1.29 10690447 PMC1724150

[32] Li F Harmer P Fisher KJ McAuley E Chaumeton N Eckstrom E. Tai Chi and fall reductions in older adults: a randomized controlled trial. J Gerontol A Biol Sci Med Sci. (2005) 60:187–94. 10.1093/gerona/60.2.187 15814861

[33] Lan C Lai JS Chen SY Wong MK. Tai Chi Chuan to improve muscular strength and endurance in elderly individuals: a pilot study. Arch Phys Med Rehab. (2000) 81:604–7. 10.1016/S0003-9993(00)90042-X 10807099

[34] Xu D Hong Y Li J Chan K. Effect of tai chi exercise on proprioception of ankle and knee joints in old people. Brit J Sport Med. (2004) 38:50–4. 10.1136/bjsm.2002.003335 14751946 PMC1724726

[35] Nelson-Wong E Poupore K Ingvalson S Dehmer K Piatte A Alexander S. Neuromuscular strategies for lumbopelvic control during frontal and sagittal plane movement challenges differ between people with and without low back pain. J Electromyogr Kinesiol. (2013) 23:1317–24. 10.1016/j.jelekin.2013.08.011 24080287

[36] Kendall KD Schmidt C Ferber R. The relationship between hip-abductor strength and the magnitude of pelvic drop in patients with low back pain. J Sport Rehabil. (2010) 19:422–35. 10.1123/jsr.19.4.422 21116011

[37] Hoffman SL Johnson MB Zou D Van Dillen LR. Sex differences in lumbopelvic movement patterns during hip medial rotation in people with chronic low back pain. Arch Phys Med Rehab. (2011) 92:1053–9. 10.1016/j.apmr.2011.02.015 21704784 PMC3124680

[38] Hall A Copsey B Richmond H Thompson J Ferreira M Latimer J. Effectiveness of tai chi for chronic musculoskeletal pain conditions: updated systematic review and meta-analysis. Phys Ther. (2017) 97:227–38. 10.2522/ptj.20160246 27634919

